# Molecular and serological evidence of hepatitis E virus genotype 3 circulation in pigs in Senegal

**DOI:** 10.1016/j.onehlt.2026.101557

**Published:** 2026-09-08

**Authors:** Bacary Djilocalisse Sadio, Mamadou Malado Jallow, Abdourahmane Sow, Sala Gaye, Toffène Diome, Moussa Dia, Aboubacry Gaye, Cheikh Momar Nguer, Marie Pedapa Mendy, Oumar Faye, Mbacké Sembène, Gamou Fall, Martin Faye, Ndongo Dia

**Affiliations:** a Virology Department, Institut Pasteur de Dakar, 220 Dakar, Senegal; b Public Health Department, PHD, Institut Pasteur de Dakar, 220 Dakar, Senegal; c Department of Public Health and Preventive Medicine, Faculty of Medicine, Pharmacy and Odonto -stomatology, University Cheikh Anta Diop of Dakar, Senegal; d Department of Animal Biology, Faculty of Sciences and Technology, Cheikh Anta Diop University, BP 5005 Dakar, Senegal; e Department of Chemical Engineering and Applied Biology, Cheikh Anta Diop University, Ecole Supérieure Polytechnique, 5085 Dakar-Fann, Senegal

**Keywords:** Hepatitis E virus, Pigs, Molecular characterization, Genotype 3, Seropositivity, Senegal

## Abstract

**Objectives:**

In Senegal, data on the epidemiology of hepatitis E virus (HEV) are limited, despite the emergence of a human epidemic in 2014. The objective of this study is to assess the prevalence, molecular characteristics, and seroprevalence of HEV in pigs in Senegal.

**Method:**

Blood samples were collected from pigs immediately after slaughter between September 2018 and December 2019. HEV RNA was detected using real-time reverse transcription polymerase chain reaction, and positive samples were subjected to Sanger sequencing targeting a fragment of approximately 300 bp of the ORF2 gene for molecular characterization. An enzyme immunoassay was performed to assess the seropositivity for anti-HEV antibody.

**Results:**

Among the 1600 serum samples tested, HEV RNA was detected in 69 serum samples (4.3%). Of these, 13 were successfully sequenced, and phylogenetic analysis showed that all strains belonged to genotype 3. Among 1161 selected RT-qPCR-negative serum tested, 76.6% were positive for anti-HEV antibodies.

**Conclusion:**

Pigs have been identified as an important reservoir of HEV genotype 3 in Senegal, highlighting the need for integrated “One Health” surveillance to better understand viral circulation and assess the potential risk of zoonotic transmission.

## 1 Introduction

The hepatitis E virus (HEV) is a major emerging public health problem worldwide [1]. It is estimated that the population living in endemic areas represents about one-third of the world's population [2], with an estimated 70,000 deaths per year [3]. This virus belongs to the *Hepeviridae* family and the *Orthohepevirus* genus [4]*.* Mortality rates associated with hepatitis E in the general population range from 0.5% to 4% [3]. One of the particularities of this virus is the very high mortality rate it causes in pregnant women. Indeed, pregnant women are disproportionately affected and are more likely to develop complicated forms of the disease, with mortality rates reaching up to 20% during the third trimester of pregnancy. This high mortality makes hepatitis E the most severe viral hepatitis infections during pregnancy [5].

To date, there are eight genotypes (HEV-1 to HEV-8) that infect humans and several domestic and wild mammals [6]. Among these, genotypes 1–4 are the most extensively studied. Thus genotypes 1 and 2 infect only humans, while genotypes 3 and 4 primarily infect mammals, with occasional transmission to humans [7].

HEV is mainly transmitted via the fecal-oral route; however, other transmission pathways have been documented, including zoonotic transmission, direct human-to-human transmission, vertical transmission, foodborne transmission, breastfeeding, and parenteral transmission [8]. Regarding zoonotic transmission, it has been associated with the consumption of raw or undercooked meat from wild boar or venison [9]. Unlike other hepatitis viruses, interspecies transmission of HEV via oral and parenteral routes has been experimentally demonstrated between porcine and human HEV strains in pigs and macaques, respectively [10]. Furthermore, rabbit HEV strain has been shown to infect pigs and to be indirectly transmitted to humans [11], [12].

Although humans are considered the primary reservoir of HEV, several, studies have demonstrated that domestic animals can also serve as reservoirs. Serological surveys in pigs, wild boars, deer, rabbits, rats, mongooses, horses, cats, dogs, sheep, goats, and cattle have revealed the presence of anti-HEV antibodies [13]. In some domestic animal populations, HEV infection rates can exceed 95% [14]. Specifically, regarding pigs, recent studies, particularly in pigs in Kathmandu, suggest that pigs may act as a reservoir for HEV during inter-epidemic periods, whereas humans are infectious mainly during the acute phase of infection. The high incidence of HEV detected in pigs during periods of low human incidence support the role of pigs as a reservoir for HEV [15].

In Senegal, a hepatitis E virus genotype 2 outbreak was reported for the first time in 2014 in the Kedougou region in Southeastern Senegal [16]. However, the source of contamination associated with this outbreak was not identified, and data on HEV circulation in the country remain limited. A study conducted in the Saint-Louis region in northern Senegal suggests that pigs may represent an important reservoir for HEV [17]. Therefore, to better understand the mechanisms underlying HEV emergence in Senegal, we conducted this study to assess the prevalence and serological evidence of HEV infection in pigs originating from eight of the fourteen regions of the country between September 2018 and December 2019, and to identify the circulating genotypes.

## 2 Methods

### 2.1 Study design and sample collection

**Fig. 1:**
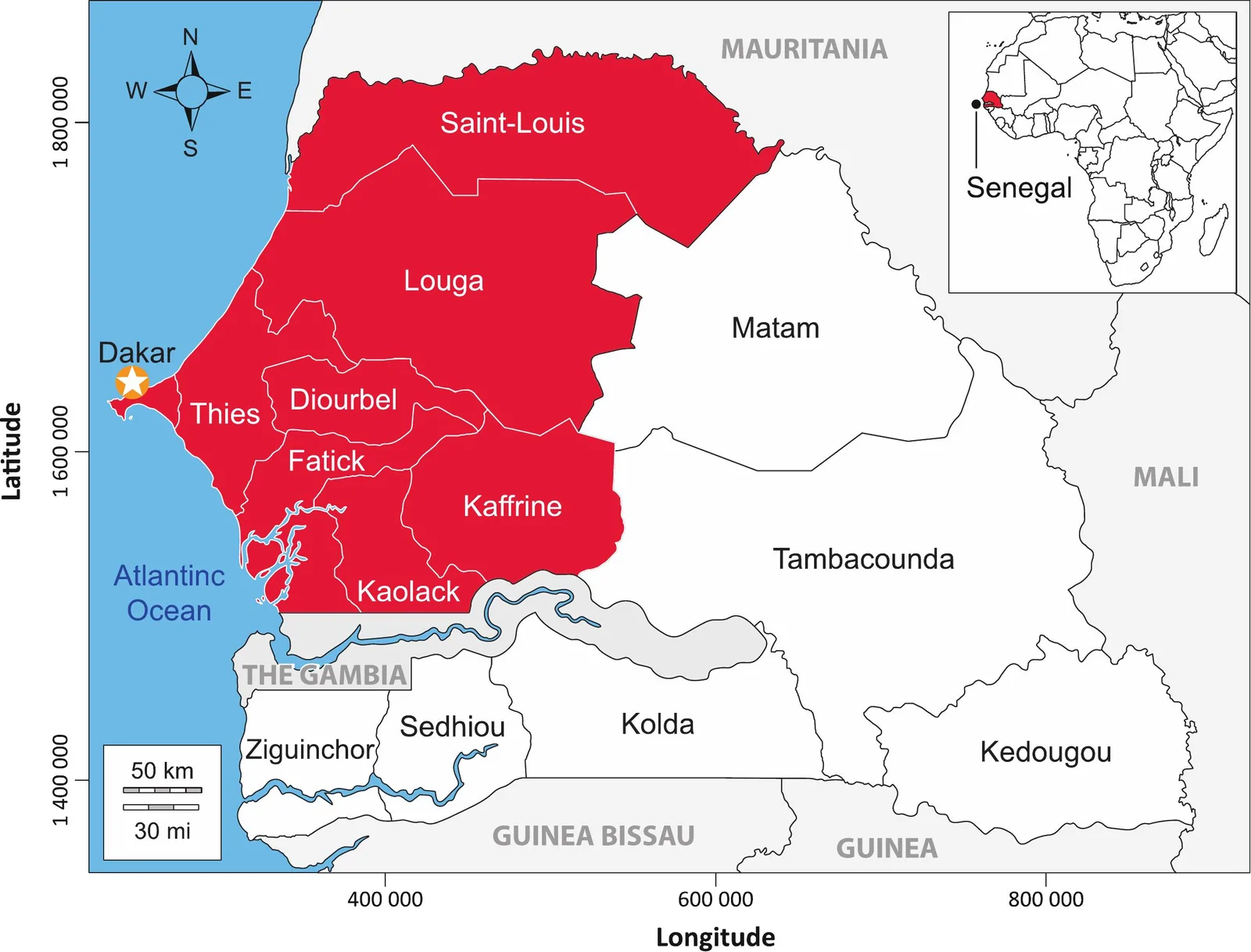
Geographical representation of the regions of origin of pigs included in the study. This figure shows a map of Senegal. The yellow and white star represents the capital. The regions included in the study are shown in red, those not included are shown in white, and Senegal's neighboring countries are shown in gray. Base maps were generated using ArcGIS Pro Desktop software (Basic license; ESRI 2025, Redlands, CA: Environmental Systems Research Institute; https://www.esri.com), then exported as Adobe Illustrator Exchange (AIX) format for further aesthetic design using Adobe Creative Cloud (version 6.9; Adobe Inc. 2025, San Jose, CA; https://www.adobe.com/creativecloud). (For interpretation of the references to color in this figure legend, the reader is referred to the web version of this article.)

This retrospective study was conducted using samples collected between September 2018 and December 2019 from pigs at the Grand Yoff slaughterhouse and pig market, a site where pigs from 8 regions of the country are temporarily fattened before slaughter for meat sale (Fig. 1). Blood samples were collected from each animal immediately after slaughter on a weekly basis, as previously described [18]. All samples were transported on the same day to the National Influenza Centre (NIC) at the Institut Pasteur de Dakar (IPD) in accordance with triple-packaging procedures. Upon arrival in the laboratory, serum was separated from clotted blood by centrifugation at 3000 rpm for 10 min and stored in newly labeled cryotubes at −80 °C until analysis. Information on the region of origin and the date of sample collection was recorded for each pig.

### 2.2 Nucleic acid extraction and molecular detection of hepatitis E virus

Total nucleic acids were extracted from 200 μL of serum using the QIAamp Viral RNA kit (QIAGEN, Valencia, CA, USA) according to the manufacturer's instructions. Extracted RNA was eluted in 60 μL of elution buffer and tested for the presence of hepatitis E virus (HEV) RNA by real-time reverse transcription PCR (RT-qPCR). Reactions were performed in singleplex using HEV-specific primers and probe with the LightMix® Kit (TIB MOLBIOL, Berlin, Germany). Briefly, each RT-qPCR reaction was carried out in a final volume of 20 μL containing 3.5 μL of nuclease-free water, 9.5 μL of LightMix, 0.8 μL of each primer (10 μM), 0.4 μL of probe (10 μM), and 5 μL of RNA template. Amplification was performed under the following cycling conditions: reverse transcription at 55 °C for 3 min, initial denaturation at 95 °C for 30 s, followed by 42 cycles consisting of denaturation at 95 °C for 3 s, and annealing/extension at 60 °C for 12 s, with fluorescence acquisition at 40 °C for 10 s. Samples were classified as HEV-RNA-positive if an amplification curve with a Ct value ≤40 was observed. The positive control consisted of RNA extracted from a laboratory-confirmed HEV-positive sample collected during the 2014 HEV outbreak in Senegal. This control was not quantified, and no standard curve was generated, as the RT-qPCR assay was used exclusively for the qualitative detection of HEV RNA. A negative control consisting of nuclease-free water was included in each run. Ct values were used only as a relative indicator of the amount of amplifiable HEV RNA, as absolute viral loads were not determined.

### 2.3 Hepatitis E virus genotyping

All samples positive for HEV by RT-qPCR were genotyped using a nested PCR approach to amplify a fragment of the open reading frame 2 (ORF2) gene, using the One*Taq* One-Step RT-PCR Kit (New England Biolabs, Ipswich, MA, USA). The first PCR was carried out in a final reaction volume of 41.5 μL containing 25 μL of OneTaq One-Step Reaction Mix (2×), 2 μL OneTaq One-Step Enzyme Mix (25×), 2 μL of forward primer HEV-F1 (10 μM; 5′-AATTATQCYCAQTAYCQRQTTQ-3′), 2 μL of reverse primer HEV-R1 (10 μM; 5′- CCCTTRTCYTQCTQMQCATTCTC -3′), 2 μL of Rnase inhibitor, 2 μL of nuclease-free water and 10 μL of RNA template. Thermal cycling conditions consisted of reverse transcription at 48 °C for 15 min, initial denaturation at 94 °C for 1 min, followed by 40 cycles of denaturation at 94 °C for 15 s, annealing at 55 °C for 30 s at 55 °C, and extension at 68 °C for 1 min, with a final extension step at 68 °C for 5 min. For the nested PCR, 2 μL of the first-round PCR product was amplified using internal primers HEV-F2 (10 μM; 5′-GTWTATGCTYTGCATWCATGGCT-3′) and HEV-R2 (10 μM; 5′-AGCCGACGAAATCAATTCTGTC-3′) using the same condition as the first PCR. Amplification products were analyzed by electrophoresis on a 1.5% agarose gel stained with 5 μL of SafeView™ Classic (Applied Biological Materials Inc., Richmond, Canada) using 1xTAE buffer. Bands corresponding to the expected amplicon size (300 bp) were excised and purified using the NucleoSpin® Gel and PCR Clean-Up (Macherey-Nagel GmbH & Co. KG, Düren, Germany) according to the manufacturer's instructions. Purified PCR products were subsequently sent to Inqaba Biotec (Pretoria, South Africa) for Sanger sequencing in both forward and reverse directions using the nested PCR primers.

### 2.4 Phylogenetic analysis of hepatitis E virus strains

For phylogenetic analyses, nucleotide sequences obtained in FASTA format were initially assembled using GeneStudio software (GeneStudio™ Pro, version: 2.2.0.0). Consensus sequences generated for each HEV sample in this study were combined with representative HEV sequences from other countries, including reference sequences of known genotypes, retrieved from GenBank (https://www.ncbi.nlm.nih.gov). Multiple sequences alignments were performed using MAFFT with the FFT-NS-2 algorithm, followed by manual editing and trimming using BioEdit v7.1.3.0. A Maximum likelihood (ML) phylogenetic tree based on partial ORF2 gene sequences was inferred using IQ-TREE v2.0.3 under the best-fit nucleotide substitution model (GTR + I + G4). The resulting tree was visualized using FigTree v1.4.4. Branch support was assessed using 1000 ultrafast bootstrap (UFBoot) replicates, with bootstrap values ≥70% considered statistically significant.

### 2.5 Serological screening for hepatitis E virus antibodies

Serum samples that were negative by RT-PCR were screened for anti-HEV antibodies using the ID Screen® Hepatitis E Indirect Multi-species ELISA kit (IDvet, Grabels, France), according to the manufacturer's instructions. This test detects total anti-HEV antibodies without distinguishing between the different antibody classes (IgG and IgM). Therefore, it cannot specifically identify recent or acute infections from past exposure. Due to a shortage of commercial ELISA kits, it was not possible to test all 1531 negative samples by RT-qPCR. Therefore, 1161 samples were selected for serological analysis using a sampling strategy designed to ensure adequate representation of each study region, given the number of kits available. Each sample was tested in duplicate. Briefly, 190 μL of dilution buffer 2 was dispensed into each well, followed by the addition of 10 μL of negative and positive controls in duplicate, and 10 μL of each test sample into the remaining wells. The microplates were covered and incubated for 45 min at 21 °C, then washed three times with at least 300 μL of wash solution. Subsequently, 100 μL of conjugate diluted 1:10 in dilution buffer 3 was added to each well and incubated for 30 min at 21 °C. After a second washing step, 100 μL of substrate was added and the plates were incubated for 15 min in the dark at 21 °C. The reaction was stopped by adding 100 μL of stop solution, and absorbance was measured at 450 nm using a microplate reader. Net optical density (OD) values were calculated by subtracting the OD of odd wells from that of even wells. The assay was considered valid when the mean net OD of the positive controls was >0.350 and when the ratio of the mean positive control OD to the mean negative control OD was ≥3. Sample results were expressed as the sample-to-positive (S/P) ratio, calculated as the ratio of the net OD of each sample to the mean net OD of the positive controls, multiplied by 100. Samples with S/*P* < 60% were considered negative, those with 60%–70% as doubtful, and those with S/*P* > 70% as positive.

### 2.6 Statistical analysis

Region-specific HEV RNA prevalence was calculated as the proportion of RT-qPCR-positive samples among the total number of pigs tested in each region. Corresponding 95% confidence intervals (95% CIs) were calculated using the Wilson score method for binomial proportions. Comparisons between HEV-seropositive and HEV-seronegative groups were conducted using the chi-square test or Fisher's exact test, as appropriate. Statistical analyses were performed using R software (version 4.4.0), and statistical significance was defined as *P* value <0.05.

## 3 Results

### 3.1 Detection of HEV and geographical distribution of HEV-positive cases

A total of 1600 serum samples collected from pigs between September 2018 to December 2019 in Senegal were analyzed. Hepatitis E virus (HEV) RNA was detected in 69 samples, corresponding to an overall prevalence of 4.3% (95% CI: 3.4–5.4%). Regional HEV RNA prevalence varied across the study areas (Table 1). The highest prevalence was observed in Thiès (25/474; 5.3%, 95% CI: 3.6–7.7%), followed by Fatick (38/818; 4.6%, 95% CI: 3.4–6.3%), Kaolack (1/22; 4.5%, 95% CI: 0.8–21.8%), Louga (2/48; 4.2%, 95% CI: 1.2–13.9%), and Diourbel (3/114; 2.6%, 95% CI: 0.9–7.4%). No HEV RNA-positive samples were detected in pigs sampled in Dakar (0/67), Kaffrine (0/13), or Saint-Louis (0/44).

**Table 1:** Regional prevalence of hepatitis E virus (HEV) RNA in pigs sampled in Senegal between September 2018 and December 2019.

Region	Pigs tested (n)	RT-qPCR positive (n)	Prevalence (%)	[95% CI]
Dakar	67	0	0	[0.0–5.4]
Diourbel	114	3	2.6	[0.9–7.4]
Fatick	818	38	4.6	[3.4–6.3]
Kaffrine	13	0	0	[0.0–22.8]
Kaolack	22	1	4.5	[0.8–21.8]
Louga	48	2	4.2	[1.2–13.9]
Saint-Louis	44	0	0	[0.0–8.0]
Thiès	474	25	5.3	[3.6–7.7]
Total	1600	69	4.3	[3.4–5.4]

95% CI = 95% confidence interval.

### 3.2 Phylogenetic analysis of detected HEV strains

**Fig. 2:**
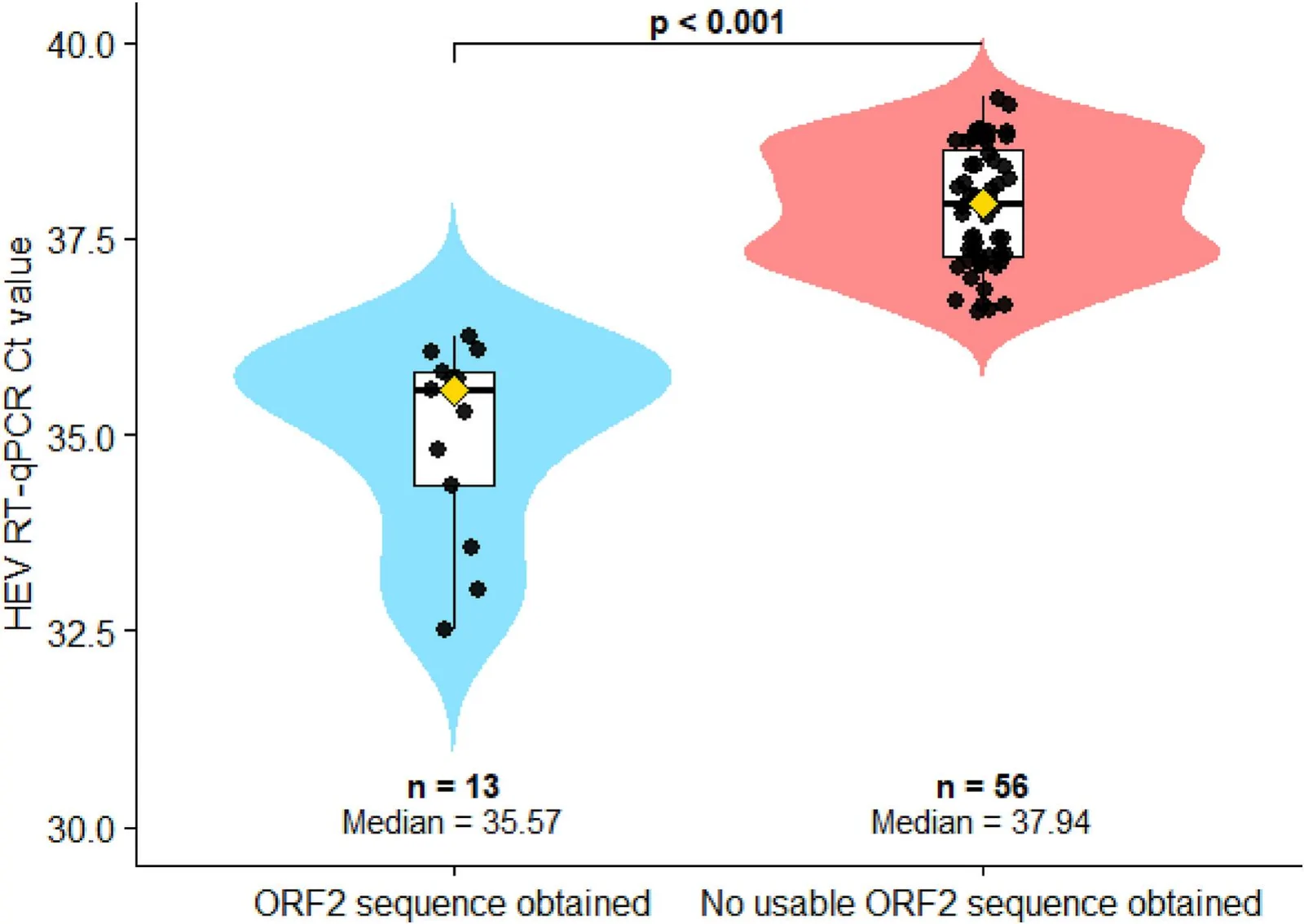
Distribution of RT-qPCR Ct values for HEV positive samples according to sequencing outcome. All 69 HEV RNA-positive samples underwent Sanger sequencing. Samples were classified as ORF2 sequence obtained (*n* = 13) when high-quality sequences were obtained, or as no usable ORF2 sequence obtained (*n* = 56) when sequencing did not provide usable sequence data. Boxes represent the interquartile range (IQR), the horizontal line indicates the median, and each point represents an individual sample. The difference in Ct values between groups was assessed using the Wilcoxon rank-sum test.

The 69 sera samples positive for hepatitis E virus (HEV) were subjected to Sanger sequencing targeting a partial fragment of the ORF2 gene. This region was selected for molecular characterization because it is a commonly used target for HEV genotyping and provides adequate phylogenetic resolution for genotype assignment while facilitating comparison with previously published HEV sequences. In total, 13 (18.8%) nucleotide sequences were successfully obtained, including 2 sequences from samples collected in 2018 and 11 from samples collected in 2019. The successfully sequenced samples had Ct values ranging from 32.52 to 36.25 (median Ct = 35.57), while those for which no usable ORF2 sequence obtained had Ct values ranging from 36.55 to 39.30 (median Ct = 37.94), these high Ct values were consistent with relatively low amounts of HEV RNA amplifiable within this assay. (Fig. 2).

**Fig. 3:**
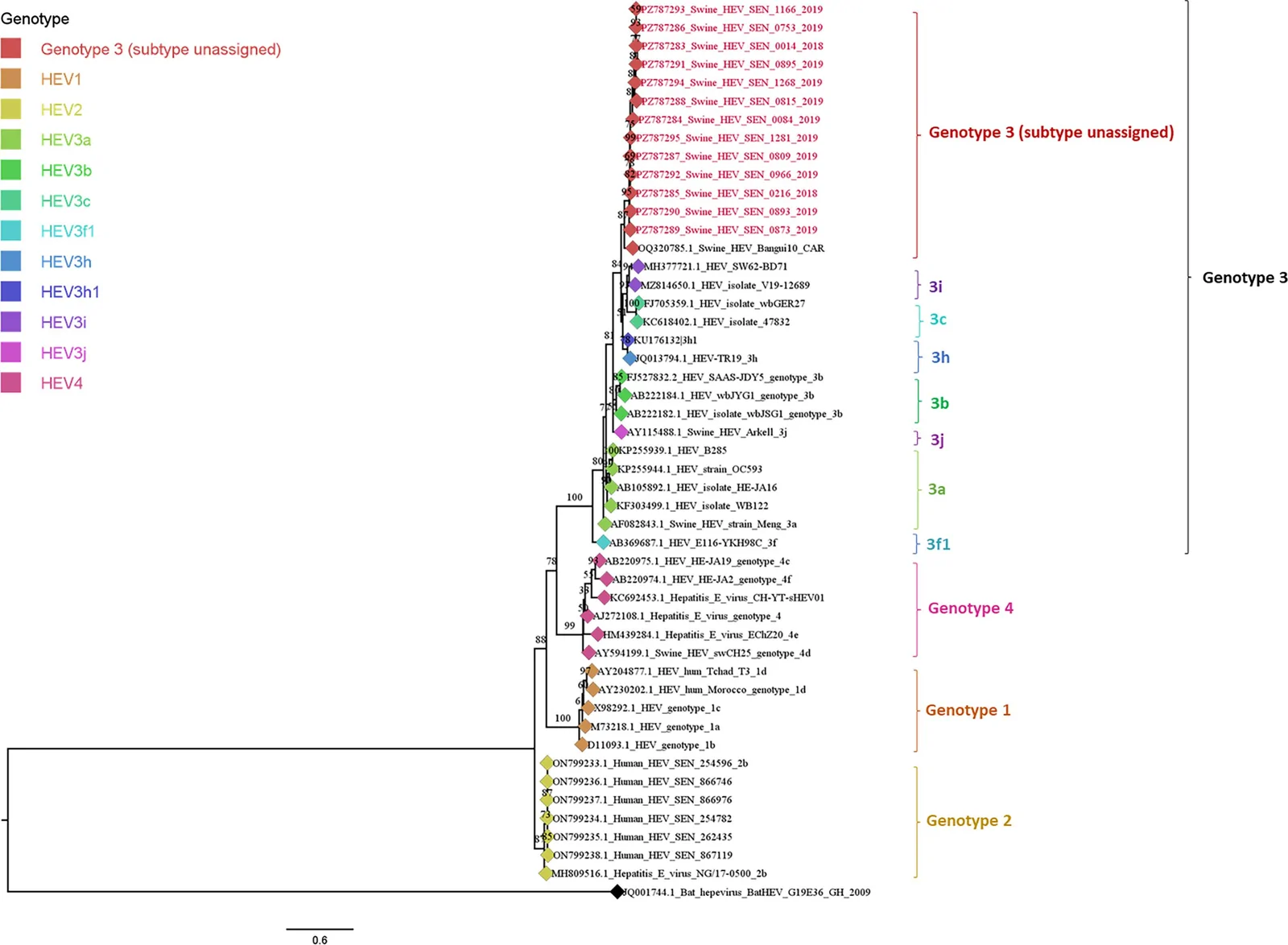
Maximum-likelihood phylogenetic tree based on 300 bp ORF2 gene nucleotide sequences of hepatitis E virus (HEV) strains obtained from pigs in Senegal. The tree was inferred using IQ-TREE version 2.0.3, under the GTR + I + G4 nucleotide substitution model, with 1000 ultrafast bootstrap replicates. The tree was visualized with FigTree (v1.4.4). Senegalese swine HEV sequences are highlighted in red. HEV sequences from human cases during the 2014 Kedougou outbreak are indicated by orange tips. (For interpretation of the references to colour in this figure legend, the reader is referred to the web version of this article.)

Phylogenetic analysis showed that all HEV strains detected in Senegalese pigs in this study formed a monophyletic group and were assigned to genotype 3, but their subtypes remained unclassified. These Senegalese strains were closely related to HEV strains previously isolated from pigs in Bangui, Central African Republic. Due to the relatively short size of the analyzed ORF2 gene fragment (∼300 bp), the phylogenetic resolution was insufficient to allow reliable assignment of strains to the different subgenotypes of genotype 3. In contrast, HEV sequences obtained from human cases during the 2014 outbreak in the Kedougou region (sequences marked with orange tips) clustered within genotype 2 and were clearly distinct from the swine HEV strains identified in this study (Fig. 3).

### 3.3 Serological evidence of HEV infection in Senegalese pigs

Among the 1531 RT-qPCR-negative serum samples, 1161 were included in the serological analysis. Sample selection was constrained by the limited availability of ELISA kits while ensuring adequate representation of all study regions. Of the 1161 samples tested, 889 were positive for anti-HEV antibodies, corresponding to an anti-HEV seropositivity of 76.6% among the selected RT-qPCR-negative samples (Table 2). Marked regional variation in HEV seroprevalence were observed; however, seropositivity rates remained consistently above 60.0% across all regions, indicating widespread circulation of HEV in the investigated pig farming areas. The lowest seroprevalence was recorded in Kaolack (63.2%), which nevertheless reflects substantial exposure to HEV. In contrast, the highest seroprevalence rates were observed in Kaffrine (90.0%), Louga (85.0%), Saint-Louis (82.9%), Dakar (81.1%), and Thiès (78.3%). In Fatick (74.8%) and Diourbel (73.6%), relatively similar seroprevalence levels were found. No statistically significant difference in HEV seroprevalence was observed between regions (*P-*value = 0.329).

**Table 2:** Seroprevalence of hepatitis E virus (HEV) infection in pigs by region in Senegal. We performed analyses to describe the population of the residential area based on the presence of HEV. A comparison between positive and negative cases was performed using the exact chi-square test. Percentages were calculated by relating the number of cases in each area to the total number of cases. *P*-values were obtained by performing comparisons using the exact chi-square statistical test. Statistical analyses were performed using R software (version 4.4.0).

	Negative (*N* = 272)	Positive (*N* = 889)	Total (*N* = 1161)	*p* value
Region				0.329
Dakar	10 (18.9%)	43 (81.1%)	53 (100.0%)	
Diourbel	23 (26.4%)	64 (73.6%)	87 (100.0%)	
Fatick	145 (25.2%)	430 (74.8%)	575 (100.0%)	
Kaffrine	1 (10.0%)	9 (90.0%)	10 (100.0%)	
Kaolack	7 (36.8%)	12 (63.2%)	19 (100.0%)	
Louga	6 (15.0%)	34 (85.0%)	40 (100.0%)	
Saint-Louis	7 (17.1%)	34 (82.9%)	41 (100.0%)	
Thiès	73 (21.7%)	263 (78.3%)	336 (100.0%)	

## 4 Discussion

In Africa, hepatitis E virus (HEV) outbreaks are increasingly reported and constitute a growing public health concern [19], [20], [21]. Between 2014 and 2025, the number of countries experiencing HEV outbreaks increased from 12 to 18. In Senegal, a major HEV outbreak occurred in 2014 and was associated with particularly high mortality among pregnant women, reaching 100% in reported cases [16]. Despite investigations conducted on rodents and water samples at the outbreak site, the source of contamination was not identified. This situation raises important questions regarding potential zoonotic reservoirs and transmission pathways of HEV in Senegal. Several studies have identified pigs as a major reservoir of HEV and a significant risk factor for zoonotic transmission [22], [23], [24], [25]. Within this context, the present study aimed to detect and molecularly characterize HEV circulating in pigs from different regions of Senegal (Kaolack, Thiès, Louga, Fatick, Diourbel, Kaffrine, Saint Louis and Dakar) in order to assess their potential contribution to HEV epidemiology in the country. An overall prevalence of 4.3% was detected in pig blood samples in this study. This prevalence is comparable to that reported by Tène et al. in Saint-Louis, where a prevalence of 5.4% was observed in pigs [6]. However, unlike the study by Tène et al., no HEV RNA was detected in pigs from the Saint-Louis region. This discrepancy may be explained by differences in sampling strategies, as Tène et al. reported higher detection rates in pig liver samples (22.2%), the primary site of HEV replication [26], compared with lower prevalence (3.1%) in meat samples. In contrast, our study exclusively analyzed blood samples, which may have reduced the likelihood of viral RNA detection. Regional variations in HEV prevalence were observed and appear to be influenced by pig farming practices and socio-cultural factors. The higher proportion of HEV RNA-positive pigs from the Thiès (5.3%, 95% CI: 3.6–7.7%) and Fatick (4.6%, 95% CI: 3.4–6.3%) regions may reflect regional differences in pig population density and husbandry practices. In Senegal, pig farming is primarily concentrated in these regions, where it is practiced almost exclusively by Christian communities. Production relies mainly on small-scale, traditional, semi-extensive systems with limited biosecurity measures. Pigs are generally raised outdoors or free-range, which increases the risks of environmental exposure and HEV transmission [27]. However, the animals included in this study were sampled at a Dakar pig slaughterhouse after being transported from different regions, and detailed farm-level information, including the production system, biosecurity conditions, and age at slaughter, was not available. Although all slaughtered pigs had reached at least weaning age (approximately 3 months), their exact age could not be determined. Since HEV viremia is most often observed in pigs aged 2 to 4 months, the lack of age-stratified data limits the interpretation of regional differences in HEV RNA detection. Future studies integrating epidemiological and detailed farm-level data are needed to better identify the factors driving HEV transmission in Senegal. The lower prevalence observed in Diourbel, Kaolack and Louga may be explained by limited pig farming activity and pigs are relatively scarce. Additionally, seasonal movements of pigs between regions, particularly during religious festivities such as Easter, may influence HEV circulation patterns. Although HEV RNA was not detected by RT-qPCR in pigs from Dakar, Kaffrine, and Saint-Louis, serological analyses revealed high HEV seroprevalence rates (>80%), indicating widespread and long-standing exposure of pigs to HEV in these regions. This discrepancy between molecular and serological findings likely reflects past infections rather than active viremia at the time of sampling.

For the molecular characterization based on a partial fragment (300 bp) of the ORF2 gene, 13 sequences were obtained from the 69 HEV positive samples. Although other genomic regions, including ORF1, have also been used for HEV genotyping and can be suitable in certain contexts, only ORF2-specific primers were available for this study. Given the high Ct values observed in most HEV RNA-positive samples, the low amounts of HEV RNA amplifiable was considered the main factor limiting sequencing success. Therefore, amplification of another genomic region such as the ORF1 gene would likely not have significantly improved the sequencing success rate. Phylogenetic analysis revealed that all HEV strains detected in pigs belonged to genotype 3. This genotype had previously been identified in pigs in Saint-Louis, Senegal [6] and has also been reported in pigs in at least ten African countries [27], [28], [29], [30], [31], as well as in several other regions of the world [32], [33], [34], [35], [36]. Although several studies have documented the co-circulation of different subgenotypes of genotype 3 in pigs [37], [38], [39], none of the sequences obtained in our study could be definitively assigned to a subgenotype. This limitation is primarily due to the relatively short size of the analyzed ORF2 gene fragment (∼300 bp), which did not provide sufficient phylogenetic resolution for reliable subgenotype classification. Therefore, sequencing of longer genomic regions or even entire genomes, would be necessary to enable robust subgenotype classification. Despite the circulation of HEV genotype 3 in pigs, human infections documented to date in Senegal have been associated with genotype 2, and no genetic link between swine and human HEV strains was identified in this study.

This study has several limitations that should be considered when interpreting the findings. First, although pigs were sampled from eight regions, these represent only part of the national territory; therefore, the results may not fully capture the geographic heterogeneity of HEV circulation in Senegal, particularly in regions with different pig production systems or ecological contexts. Second, molecular detection of HEV was performed only on serum samples. Since viremia is generally transient during infection, this approach may have underestimated the true prevalence of active infection. Testing additional sample types such as feces, liver, or bile, known to contain higher viral loads [40], [41], would likely have improved detection sensitivity. Third, the anti-HEV seropositivity reported in this study should be interpreted in light of the study design. Owing to a shortage of commercial ELISA kits, serological testing was limited to a subset of RT-qPCR-negative samples selected to ensure adequate representation of all study regions. Consequently, the reported seropositivity does not represent the overall seroprevalence among all pigs included in the study but rather the seropositivity among the selected RT-qPCR-negative animals. Although the sampling strategy was designed to maintain regional representativeness, some degree of selection bias cannot be excluded and should be considered when interpreting the findings. Furthermore, the ID Screen® Hepatitis E indirect multispecies ELISA test detects total anti-HEV antibodies but does not distinguish between IgG and IgM. Consequently, it cannot differentiate between recent or acute infection and prior exposure. Combining molecular detection with an HEV-specific IgM serological test would have allowed for a more comprehensive characterization of the infection stage and the relationship between the immune response and viremia. Finally, despite the relatively high number of RT-PCR positive samples (*n* = 69), only a limited subset (*n* = 13) could be successfully characterized, likely due to low viral loads or RNA degradation. This restricted sequencing success, combined with the use of a short partial ORF2 fragment (∼300 bp), limits the representativeness and phylogenetic resolution of the molecular data and may have led to an underestimation of the genetic diversity of HEV strains circulating in pigs in Senegal. Consequently, sequencing longer genomic regions, or even entire genomes, would allow for a better characterization of the genetic diversity and transmission dynamics of HEV in Senegal. Nevertheless, despite these limitations, our findings confirm that pigs constitute an important reservoir of hepatitis E virus genotype 3 in Senegal and underscore the need for integrated One Health surveillance to monitor viral evolution and assess the potential emergence of zoonotic HEV strains.

## 5 Conclusion

This study provides comprehensive molecular and serological evidence of hepatitis E virus (HEV) circulating in pigs in Senegal. Although the prevalence of HEV was relatively low (4.3%), most positive cases were detected in regions with high pig farming density, such as Fatick and Thiès, suggesting that production intensity may influence viral circulation. Phylogenetic analysis demonstrated that all successfully characterized strains belonged to genotype 3 and were genetically distinct from the genotype 2 strains responsible for the 2014 human outbreak in southeastern Senegal (Kedougou), indicating the circulation of different HEV genotypes in the country. Furthermore, the high seropositivity rates observed in pigs across all sampled regions indicate widespread exposure to HEV within the swine population. Taken together, these findings identify pigs as an important reservoir of HEV genotype 3 in Senegal and highlight the need for integrated One Health surveillance to better understand viral transmission dynamics and assess the potential risk of zoonotic HEV infections.

## CRediT authorship contribution statement

**Bacary Djilocalisse Sadio:** Writing – review & editing, Writing – original draft, Validation, Methodology, Investigation, Formal analysis, Data curation, Conceptualization. **Mamadou Malado Jallow:** Writing – review & editing, Writing – original draft, Investigation, Formal analysis, Data curation. **Abdourahmane Sow:** Writing – review & editing, Writing – original draft, Resources, Investigation, Formal analysis. **Sala Gaye:** Writing – review & editing, Methodology, Data curation. **Toffène Diome:** Writing – review & editing, Supervision. **Moussa Dia:** Writing – review & editing. **Aboubacry Gaye:** Formal analysis. **Cheikh Momar Nguer:** Writing – review & editing, Supervision. **Marie Pedapa Mendy:** Methodology. **Oumar Faye:** Writing – review & editing, Validation, Supervision, Resources. **Mbacké Sembène:** Writing – review & editing, Validation. **Gamou Fall:** Writing – review & editing, Visualization, Validation, Supervision. **Martin Faye:** Writing – review & editing, Writing – original draft, Visualization, Validation, Supervision. **Ndongo Dia:** Writing – review & editing, Writing – original draft, Visualization, Validation, Supervision, Resources.

## Ethical approval

The samples analyzed in this study were collected as part of an ongoing animal influenza surveillance program coordinated by the Institut Pasteur de Dakar in collaboration with the Senegalese veterinary authorities. Blood samples were collected from pigs slaughtered in approved abattoirs during routine commercial meat production and slaughterhouses and were subsequently used for secondary analyses, including hepatitis E virus detection. No animals were specifically sacrificed for research purposes. Sampling was carried out in accordance with the guidelines of the Senegalese Ministry of Agriculture, Food Sovereignty, and Livestock governing animal health surveillance. Prior to sampling, the objectives and procedures of the surveillance program were explained to veterinarians and pig farmers, who provided oral informed consent. Sampling was performed by trained professional veterinarians and zoologists, and all procedures complied with current national regulations on animal health, food safety, and biosecurity.

## Ethics declaration

This study was conducted in accordance with the following guidelines for animal welfare and/or reporting: Sampling was conducted in accordance with the guidelines of the Senegalese Ministry of Agriculture, Food Sovereignty, and Livestock governing animal health surveillance, as well as applicable national regulations on animal health, food safety, and biosecurity.

Ethics committee approval was not required. Ethics approval was not required because the study involved secondary analysis of blood samples collected through an ongoing animal influenza surveillance program. The samples were obtained from pigs slaughtered in approved abattoirs as part of routine commercial meat production, and no animals were specifically sacrificed or handled for the purposes of this study. Sampling was conducted in accordance with applicable national regulations and guidelines governing animal health surveillance, food safety, and biosecurity in Senegal.

## Funding source

This research received no specific grant from any funding agency in the public, commercial, or not-for-profit sectors and was only supported by the 10.13039/501100003762Institut Pasteur de Dakar internal funds for research.

## Declaration of competing interest

The authors declare that they have no competing interests.

## Data Availability

The nucleotide sequence data generated and analyzed during this study are publicly available in the GenBank database under accession numbers PZ787283 to PZ787295.
